# Assessing the synergistic effectiveness of intermittent theta burst stimulation and the vestibular ocular reflex rehabilitation protocol in the treatment of Mal de Debarquement Syndrome: a randomised controlled trial

**DOI:** 10.1007/s00415-024-12215-5

**Published:** 2024-02-12

**Authors:** Cherylea J. Browne, S. R. Sheeba, T. Astill, A. Baily, C. Deblieck, V. Mucci, R. Cavaleri

**Affiliations:** 1 School of Science, Western Sydney University, Sydney, NSW Australia; 2 Brain Stimulation and Rehabilitation (BrainStAR) Laboratory, Western Sydney University, Sydney, NSW Australia; 3 Translational Neuroscience Facility, School of Medical Sciences, UNSW Sydney, Sydney, NSW Australia; 4 Western Sydney University, Translational Health and Research Institute, Sydney, NSW Australia; 5 School of Health Sciences, Western Sydney University, Sydney, NSW Australia; 6 Laboratory of Equilibrium Investigations and Aerospace (LEIA), University of Antwerp, Antwerp, Belgium

**Keywords:** Mal de Debarquement, Mal de Debarquement Syndrome, MdDS, Vestibular, Neuro-otology, Theta burst stimulation, Vestibular rehabilitation

## Abstract

**Introduction:**

Mal de Debarquement Syndrome (MdDS) is a rare central vestibular disorder characterised by a constant sensation of motion (rocking, swaying, bobbing), which typically arises after motion experiences (e.g. sea, air, and road travel), though can be triggered by non-motion events. The current standard of care is non-specific medications and interventions that only result in mild-to-moderate improvements. The vestibular ocular reflex (VOR) rehabilitation protocol, a specialised form of rehabilitation, has shown promising results in reducing symptoms amongst people with MdDS. Accumulating evidence suggests that it may be possible to augment the effects of VOR rehabilitation via non-invasive brain stimulation protocols, such as theta burst stimulation (TBS).

**Methods:**

The aim of this randomised controlled trial was to evaluate the effectiveness of intermittent TBS (iTBS) over the dorsolateral prefrontal cortex in enhancing the effectiveness of a subsequently delivered VOR rehabilitation protocol in people with MdDS. Participants were allocated randomly to receive either Sham (*n* = 10) or Active (*n* = 10) iTBS, followed by the VOR rehabilitation protocol. Subjective outcome measures (symptom ratings and mental health scores) were collected 1 week pre-treatment and for 16 weeks post-treatment. Posturography (objective outcome) was recorded each day of the treatment week.

**Results:**

Significant improvements in subjective and objective outcomes were reported across both treatment groups over time, but no between-group differences were observed.

**Discussion:**

These findings support the effectiveness of the VOR rehabilitation protocol in reducing MdDS symptoms. Further research into iTBS is required to elucidate whether the treatment has a role in the management of MdDS. TRN: ACTRN12619001519145 (Date registered: 04 November 2019).

## Introduction

Mal de Debarquement Syndrome (MdDS) is a rare central vestibular disorder characterised by a constant sensation of non-spinning vertigo (i.e. rocking, swaying, and bobbing) [1–3]. These motion sensations are commonly accompanied by a range of symptoms such as imbalance, ‘brain fog’, visual induced dizziness, sensitivity to light and sounds, anxiety, depression, and migraine [4–9]. MdDS is typically triggered by exposure to passive motion, like that experienced on a boat, airplane, or motor vehicle. While some degree of non-spinning vertigo is common and transient following such experiences (termed Mal de Debarquement), a diagnosis of the chronic form, MdDS, requires symptoms to persist for at least one month [3, 5, 10]. Not all onsets of MdDS can be attributed to a passive motion event, and a small subset of patients associate the onset of their symptoms to non-motion events, such as intense stress, sickness, or childbirth, and some individuals cannot identify any triggering event at all. The differing onset types have been classified as motion-triggered (MT) and non-motion-triggered (NMT) MdDS, respectively [3–5, 11, 12]. The MdDS clinical population demonstrates a female/male ratio of between 8:2 and 9:1 [4, 5, 8, 10, 13]. In females, the age of MdDS onset is commonly between 40 and 50 years, whereas male exhibits greater variability in age of onset [4, 5, 8, 10, 13]. Previously, poor diagnostic criteria and lack of understanding of the condition in the medical community resulted in a high rate of misdiagnosed or undiagnosed patients [5]. Though more recent diagnostic guidelines have become available [3], diagnosis is still complicated by an overlapping of symptoms with other vestibular pathologies, such as persistent postural perceptual dizziness [14] and vestibular migraine [15]. The underlying pathogenesis of MdDS is unknown, which limits treatment options. Currently, standard care for people with MdDS is benzodiazapines and other anti-anxiety or anti-depressant medications, such as selective serotonin reuptake inhibitors [4, 8, 16]. Unfortunately, the effectiveness of these medications is mixed, from no reduction to only moderate reduction of symptoms, and none are considered to be curative [16]. Another drawback of these types of medications is their potential for addiction [8] and the development of tolerance [17], limiting prolonged usage. Other non-specific treatments, such as standard vestibular rehabilitation, chiropractic treatments, vestibular suppressants, and counselling, have been trialed with no substantial benefits [12, 16, 18, 19].

Given the limited utility and negative sequalea associated with pharmacological interventions, increasing emphasis has been placed upon the need for non-pharmacological strategies to target the symptoms of MdDS [8, 16, 17]. One intervention that has shown promise is MdDS-specific vestibular rehabilitation [20, 21], based on recalibrating the vestibular ocular reflex (VOR), a neurological reflex which maintains ocular stability by generating compensatory eye movements during head movement [22]. It has been proposed that MdDS is the result of VOR maladaptation involving the central integrative mechanism in the vestibular system, the velocity storage (VS), and a multisensory element that modulates the time constant of the VOR with respect to that of semicircular canal afferents [21]. Evidence of VOR maladaptation in humans has been demonstrated in NASA space flight experiments [23], where participants developed oscillating vertical nystagmus on head movement following prolonged exposure to a slowly rotating room [23]. Yakushin and colleagues confirmed that MdDS patients similarly have longer VOR time constants compared to age-matched controls, suggesting VOR maladaptation [24]. To target this phenomenon, Dai and colleagues pioneered the VOR rehabilitation protocol [21], with the aim to recalibrate the VOR by exposing the patient to full-field horizontal or vertical optokinetic (OKN) stimuli (stripes) (moving in the opposite direction of the patient’s rotation or “gravitational pull”) coupled with passive head movements, in order to induce changes in the VS [21]. In their experiments, 24 individuals with MdDS were treated with a 5-day unstandardised VOR rehabilitation protocol. While improvements were observed, approximately 1 in 3 people did not have complete or substantial recovery 1 year post-treatment [21]. Similar findings have been reported in subsequent research [20, 25]. The later studies also reported that the VOR rehabilitation protocol had a better response rate in those with MT MdDS compared to those with NMT MdDS. Additionally, despite improvements in non-spinning vertigo perception, remission was rare and residual symptoms, such as high visual sensitivity, migraine-like symptoms, and brain fog, were common post-treatment [20, 25]. This suggests that there is a need for studies exploring avenues to augment the effectiveness of the VOR rehabilitation protocol.

Another non-pharmacological strategy to target the symptoms of MdDS is neuromodulation, which is based off the theory that MdDS is a disorder of neuroplasticity [10]. It was theorised that areas of the brain responsible for unconscious balance control develop an internal representation of the external environment, i.e. the persistent background oscillations of the passive motion experience [10]. An exploratory functional brain-imaging study on MdDS patients measuring brain glucose metabolism found that MdDS patients displayed hypermetabolism in the left entorhinal cortex (EC) and amygdala and displayed hypometabolism in the left prefrontal (including the dorsolateral prefrontal cortex [DLPFC]) and temporal cortex, along with the right amygdala [26]. Then, functional connectivity measurements revealed increased connectivity between the EC and sensory processing areas located in the parietal and occipital lobes and decreased connectivity with the prefrontal/premotor cortex (frontal eye field, middle frontal gyrus). MdDS subjects also exhibited reduced connectivity between homologous structures in the prefrontal cortex [26]. The changes identified in the EC are of significant interest given its key role in mediating hippocampal-neocortical communication [27–29], and spatial mapping, navigation, and cognition [30]. Another study, on individuals with transient (i.e. non-chronic) Mal de Debarquement, also measured brain glucose metabolism and found hypermetabolism in the super occipital gyrus, superior frontal gyrus, and superior and inferior parietal lobules, and hypometabolism in various cerebellar structures (i.e. inferior semi-lunar lobule, nodule, uvula and tonsil) [31]. These findings differ from the hypermetabolism and hypometabolism identified in patients with the chronic form of MdDS, though this may suggest that metabolic activity in the various parts of the brain change over time in people with MdDS, particularly between the transient experience and the chronic form. A morphometry study [32] identified duration of illness-dependent grey matter volume changes in visual-vestibular processing areas, default mode network structures, salience network structures, somatosensory network structures, and a structure within the central executive network (the DLPFC) [32]. Based on hypometabolism and grey matter changes identified in the DLPFC of people with MdDS [26, 32], this area has been the most common cortical target during neuromodulation investigations. Repetitive transcranial magnetic stimulation (rTMS), a form of non-invasive brain stimulation, targeting DLPFC has demonstrated promising short-term improvements among individuals with MdDS [33–38]. Studies of resting state functional magnetic resonance imaging and electroencephalography in people with MdDS have indicated that DLPFC rTMS decreases functional connectivity between the left EC and the precuneus, right inferior parietal lobule, and the contralateral EC [39, 40], which are part of the posterior default mode network. This reduction in connectivity has been correlated with improved MdDS symptoms [41]. Further, the DLPFC has direct projections to main cortical oculomotor areas and several areas in the posterior parietal cortex responsible for gaze stability and oculomotor control [42, 43]. Spatial information received by the posterior parietal lobes also projects to the DLPFC, making it an important area for cognitive control over spatial information processing. This is pertinent to people with MdDS who, along with rocking dizziness, experience poor attention and significant intolerance to visual motion [33]. Additionally, it has been hypothesised that the DLPFC has indirect projections to the vestibular nuclei [43], which are known to be an integral part of the reflex arc of the VOR [44]. Given the potential link between the VOR and DLPFC, this represents a promising means by which to enhance outcomes associated with the VOR rehabilitation protocol. Prior stimulation of DLPFC may therefore enhance the tolerability and effectiveness of subsequently delivered VOR protocols. Stimulation of the left DLPFC has also been shown to effectively treat symptoms of anxiety and depression [45–47], which commonly develops in people with MdDS [3–5].

While valuable, traditional rTMS is time-consuming. This is a pertinent point when treating clinical populations and given that rTMS of DLPFC has been associated with increased procedural discomfort compared to stimulation of other cortical sites [48]. Recently, theta burst stimulation (TBS) protocols have been developed, which have been shown to produce similar clinical effects to rTMS but at a fraction of the time required [49]. A previous TBS study targeting various brain areas in those with MdDS suggests that the treatment has promise, but demonstrates only modest improvements when used in isolation [50]. However, it is known that non-invasive brain stimulation alters cortical excitability and has the potential to enhance synaptic plasticity and promote synaptogenesis, providing a strong neurophysiological rationale for its use as a means of enhancing responsiveness to subsequent treatments [51]. Thus, TBS may act synergistically with subsequently delivered VOR protocols to promote neuroplastic changes and enhance patient outcomes.

The aim of this study was to evaluate the effectiveness of the VOR protocol with and without iTBS pre-treatment on objective and subjective outcomes in people with MdDS. We hypothesised that participants who receive a pre-treatment of active iTBS prior to the VOR rehabilitation protocol would demonstrate greater improvements in balance, a greater reduction of symptoms, and significant improvements in mental health scores, compared with those receiving sham iTBS prior to the VOR rehabilitation protocol.

## Methodology

### Ethical approval and trial registration

Ethical approval was provided by the local institutional Human Ethics Committee (H13563) and the trial was registered on the Australian New Zealand Clinical Trials Registry (https://www.anzctr.org.au/—Trial Id: ACTRN12619001519145) on November 4, 2019. Each respondent gave written informed consent. All investigations were conducted according to the principles expressed in the Declaration of Helsinki.

### Study population and recruitment

People with MdDS, formally diagnosed by medical specialists were recruited between December 2019 and July 2022 for the study. Patients were recruited across Australia via two Facebook pages—the MdDS Australia Support Group and the Western Sydney University MdDS Research Group.

#### Eligibility criteria

Participants were required to be reporting symptoms consistent with MdDS diagnosis guidelines [3] and have an official MdDS diagnosis from an Australian medical specialist (medical certificate or email confirmation from the specialist was required), this included Neurologists, Otolaryngologists, Ear, Nose and Throat Specialists, and Primary Care Physicians—all registered under The Australian Health Practitioner Regulation Agency (AHPRA). Participants were also required to be > 18 years old and have no contraindications to brain stimulation according to the TMS Adult Safety Screen questionnaire [52, 53]. MT and NMT onset were considered for this study.

### Group allocation

Suitable participants were randomly allocated to one of two study groups by an independent researcher using a random number generator before testing on Day 1: Active Group: Active iTBS + VOR protocol (*n* = 10), and Sham Group: Sham iTBS + VOR protocol (*n* = 10). Allocation was concealed using consecutively numbered opaque envelopes that were opened during the participant’s first testing session.

### Experimental protocol

One week prior to treatment, participants were required to complete a daily pre-treatment symptom diary at the end of each day (seven entries total). On Day 1 of the treatment week, participants were required to complete a series of mental health questionnaires and posturography tests (pre-treatment baseline). Participants then received active or sham iTBS, followed by a VOR rehabilitation protocol treatment session, after a 20-min break. An end of day posturography measurement was then recorded. On Days 2, 3 and 4, participants started with their allocated active or sham iTBS protocol, followed by up to five treatment sessions of the VOR rehabilitation protocol. End of day posturography measurements were recorded. In addition, at the end of Day 4, participants were required to complete a series of mental health questionnaires and posturography tests (post-treatment measurements). Throughout the treatment week, participants were required to have a 30-min walk around campus immediately after the end of day posturography measures were recorded, complete a daily symptom diary, at the end of the day, and were required to complete a symptom diary entry at the following post-treatment timepoints: Week 1, 2, 3, 4 and 16, at the end of the day. Participants also completed the mental health questionnaires at Week 4 and 16 post-treatment timepoints, at the end of the day. See Table 1 for more details.

**Table 1:** Clinical trial design

Pre-treatment	Treatment week				Post-treatment (in weeks)				
Day 1—7	Day 1	Day 2	Day 3	Day 4	1	2	3	4	16
MSQ (daily)	All quest.				MSQ	MSQ	MSQ	All quest.	All quest.
	Posturography								
	iTBS	iTBS	iTBS	iTBS					
	Up to 1 × VOR	Up to 5 × VOR	Up to 5 × VOR	Up to 5 × VOR					
	Posturography	Posturography	Posturography	Posturography					
	30-min walk	30-min walk	30-min walk	30-min walk					
	MSQ	MSQ	MSQ	All quest.					

This table highlights the time points for theta burst stimulation sessions, vestibular ocular reflex rehabilitation protocol sessions, and subjective and objective measurements throughout the clinical trial

*MSQ *  MdDS Symptom Questionnaire (taken at every time point), *All quest.* All questionnaires (MSQ + Hospital Anxiety and Depression Score + Dizziness Handicap Inventory + Beck Depression Inventory), *VOR* Vestibular Ocular Reflex (direction of stripes and head movements determined by preceding Fukuda Stepping Test), i*TBS* Intermittent Theta Burst Stimulation (Active or Sham)

### Questionnaires

#### Pre-trial screening forms

All participants were required to complete two paper-based forms: (1) an MdDS Clinical Data and Intake form, which included demographic details, MdDS diagnosis details, onset cause and symptom experience, menopause experience (for females only), medication usage and diagnosed medical conditions (besides MdDS), and (2) a Non-invasive Brain Stimulation screening form [52, 53], which included questions regarding medical history, current medications and seizure risk. Participants were not allowed to have taken neuroactive medications or drugs for 30 days prior to participating in the trial. These were used determine the suitability of the participant to participate in the clinical trial.

#### In-trial questionnaires

Participants were required to complete four different subjective questionnaires at various timepoints before, during and after the treatment week (see Table 1). Participants were required to complete: (1) An MdDS Symptom Questionnaire (created for this study), which collected data regarding overall MdDS symptom levels, and primary and associated symptom experience, (2) The Hospital Anxiety and Depression Scale (HADS) [54], which assesses anxiety and depression levels, (3) The Dizziness Handicap Inventory (DHI) [55], which evaluates the degree of handicap experienced in the daily lives of patients with dizziness (subdivided into three categories, functional, emotional, and physical handicap), and (4) The Beck Depression Inventory (BDI) [56], which evaluates the severity of depression. The HADS, DHI and BDI are all validated questionnaires, which have been used in multiple studies assessing these outcomes in people with MdDS and other vestibular disorders [5, 31, 36, 57–59]. The questionnaires were distributed online using Qualtrics XM (Qualtrics International Inc.). To prevent non-responses, every question required a response before the participant could proceed. Participants received reminders via email and text messages to ensure questionnaire completions for each timepoint.

### Posturography measurements

Posturography measurements were recorded (Day 1 (pre-treatment), end of Day 1, 2, 3 and 4) with the use of a Wii Balance Board (Nintendo Co., Ltd). The board has been shown to be a reliable tool in measuring postural sway, centre of pressure distance and sway velocity rate in people with vestibular disorders [20, 60–62]. BrainBLOX software was used to acquire posturography data. This software was developed in The Neuromechanics Laboratory Department of Integrative Physiology at University of Colorado Boulder, and provided an interface to capture, record, and visualise data provided by the Wii Balance Board [63]. After acquisition, the data was processed in MATLAB (Release 2017b, developed by The MathWorks, Inc., Natick, Massachusetts, USA).

Posturography was measured from the participants during four different stance tests: (1) eyes open/feet apart (hip width), (2) eyes closed/feet apart (hip width), (3) eyes open/feet together, and (4) eyes closed/feet together. During these tests, the participants were required to remain on the board for 60 s. Participants wore noise-reducing earmuffs, were barefoot with arms relaxed by their side, and directed to stare at a fixed point (for the ‘Eyes open’ tests only). On Day 1 (baseline—before any treatment) and Day 4 (Final—after both iTBS and VOR sessions), posturography from all four stance tests was recorded. At the end of Day 1, 2, and 3, only the posturography from the eyes closed/feet together stance test was recorded. For further details, see Table 1.

The posturography outcomes that were acquired by the BrainBLOX software were: the confidence ellipse area (CEA), which has been widely used for assessing posture using anterior, posterior, medial, and lateral coordinates of the centre of pressure (CoP) [20, 64–66], the area under the curve—medial/lateral (AuC_ML) and anterior/posterior (AuC_AP), which is the area under the curve of a power spectrum, with AuC_ML measuring medial and lateral sway, and AuC_AP measuring anterior and posterior sway [20, 65], distance (DIS), which is the total path length of the CoP movements [20, 65], and the velocity (VEL), which is the mean velocity of the CoP [20, 64, 65].

### Theta burst stimulation

#### Electromyography

Bipolar surface electrodes were used to record electromyographic (EMG) activity (Ag–AgCl, Noraxon dual electrodes, interelectrode distance 2.0 cm). The active electrode was placed over the belly of the right FDI, and the ground electrode was placed over the ipsilateral olecranon. Electromyographic signals were amplified (32,000), band-pass-filtered (20–1000 Hz), and sampled at 2 kHz using a Power 1401 Data Acquisition System and Signal3 software (Cambridge Electronic Design, Cambridge, UK). Participants were seated comfortably with their head supported and their right arm placed in forearm pronation and elbow flexion on a pillow across their lap.

#### Theta burst stimulation protocol

Intermittent TBS (iTBS) was applied using a Magstim Super Rapid^2^ Plus^1^ and a 70-mm air-cooled figure-of-eight coil. Biphasic stimuli were delivered with the handle placed tangentially to the skull and pointing postero-laterally at 45°, inducing a posterior-lateral to anterior-medial second phase current [67–69]. The coil was positioned over the left DLPFC in accordance with BeamF3 algorithm (clinicalresearcher.org/software.htm). A fixed, multi-hinge, TMS coil armature was used to maintain accurate coil positioning throughout the procedure. The coil location and orientation were monitored throughout the session using the Brainsight neuronavigation system. On every treatment day, participants received five ‘blocks’ of iTBS, each separated by 10-min (3000 total pulses per day). During active iTBS, bursts of three pulses were delivered at 50 Hz, repeated at 200 ms intervals in trains of 2 s. The 2 s trains of iTBS were repeated every 10 s for a total of 600 pulses per block. Each session was therefore approximately 1 h, 16-min of dedicated stimulation. This ‘excitatory’ protocol was selected as high frequency (excitatory) rTMS over the left DLPFC has previously been shown to decrease motion perception and induce reductions in long-range intrinsic functional connectivity (correlating with symptom improvement) in people with MdDS [33, 34]. Stimuli were delivered at 90% of the resting motor threshold (rMT) determined during the first treatment day [70, 71]. Resting motor threshold was defined as the minimum intensity at which 5 out of 10 stimuli, delivered to the “hotspot” of the first dorsal interosseous muscle (FDI) representation, evoked a peak-to-peak motor evoked potential (identified using electromyography) of at least 0.05 mV in the resting muscle [72]. The hotspot was defined as the coil position, identified using a Brainsight neuronavigation system (Rogue Research, Inc, Quebec, Canada), that evoked a maximal peak-to-peak motor evoked potential in the target muscle at a given stimulation intensity [72].

For sham iTBS, a sham coil (Magstim Co. Ltd, Dyfed, UK) was positioned in the same location as the active coil [73]. The sham coil replicated the audible clicking and somatic scalp sensation experienced with active iTBS [73], but did not stimulate the cortical tissue. Both the intervention (Active) and control (Sham) group received identical information and instructions. At the end of the experiment, the integrity of participant blinding was determined by asking whether participants believed they had received either active or sham iTBS.

### Vestibular ocular reflex rehabilitation protocol

During the VOR rehabilitation sessions, the participants were seated in a chair in a darkened optokinetic (OKN) cylindrical chamber, custom-built for this study. For vertical stripes, a full-field OKN visual stimulus was projected on the semi-circular wall, which filled the whole peripheral visual field of the participant, as per the studies of Dai [21, 25], and Mucci [20]. Participants were seated in the centre of the chamber, ~ 60 cm from any of the walls. During the treatment, the OKN stripes moved (either left or right) at a speed of 10°/s. The participants were instructed to stare passively at the chamber wall directly in front of them, a fixation point (in this study, a small round sticker was placed on the chamber wall) was used for those who were unable to do this [65, 74]. The participant’s head was moved at a constant frequency of 0.165 Hz [20] by the researcher with the help of a metronome. For horizontal stripes, a full-field OKN visual stimulus was projected on a blank, flat wall, using an ultra-short throw projector (Dell S500wi, Dell Inc.) which filled the whole peripheral visual field of the participant as per Mucci’s study [20]. Participants were standing ~ 60 cm from the wall. During the treatment, the OKN stripes moved (either upwards or downwards) at a speed of 10°/s. The participants were instructed to stare passively at the chamber wall directly in front of them, a fixation point (in this study, a small round sticker was placed on the chamber wall) was used for those who were unable to do this [65, 74]. The participant’s head was not moved in this setting. The OKN visual stimulus was generated by the ‘OKN Stripes Visualization Web Application’ [75] available online. Settings were as follows: direction of movement – ‘bottom to top’ or ‘top to bottom’, number of stripes—28, speed—20).

During the four days of VOR treatment, participants underwent up to five sessions per day, each session lasting for up to 4 min, with the exception of Day 1, where only one shortened OKN stimulation was delivered to acclimate the participant and ensure they did not experience any negative side effects (see Table 1). A 10–20-min interval was provided between each session of OKN stimulation. The participants had the right to stop at any moment.

#### Determination of direction of stripes and head movement

The direction of the OKN stripes was determined by one of three main variables—the Fukuda Stepping Test (FST), the tandem stance balance test or the description of the patient’s perception of internal oscillation. For the Fukuda Stepping Test, participants marched on the spot for 45 s while wearing noise-reducing earmuffs, barefoot, with eyes closed and arms held out straight [76]. The direction of participant rotation during the test was used to determine the direction of the OKN stripes, which were set to move in the opposite direction to the participant’s rotation. If the participant did not rotate during the FST, which was rare (< 5% of the FST results), the tandem stance balance (Sharpened Romberg) test results were used, which involved the participant standing with their left foot directly in front of their right, followed by standing with their right foot directly in front of their left, each for 30 s [77, 78]. This was done barefoot, with eyes closed, and while wearing noise-reducing earmuffs. If the participant displayed a clear direction of lean or stepping to one side to ‘catch’ themselves, the stripes were set for the opposite direction. If this test did not show a clear result, the participant’s perception of internal oscillation was used to determine the direction of the stripes. This was performed prior to every VOR session. Based on the results of one of these three main variables, the direction of the stripes were as follows: when the participant rotated, leant or perceived pulling to the right = vertical stripes were moved from right to left, when the participant rotated, leant or perceived pulling to the left = vertical stripes were moved from left to right, when the participant marched, leant or perceived pulling forwards = horizontal stripes were moved from bottom to top, and when the participant marched, leant or perceived pulling backwards = horizontal stripes were moved from top to bottom [20].

The type of head movement was determined by the participant’s primary motion sensation experience. If the participant primarily experienced bobbing (up-and-down motion sensation, commonly described as bouncing, cloud walking, etc.) then the participant’s head was manually moved up and down, resulting in the same movements produced by extension and flexion of the cervical vertebrae (i.e. chin moving upwards away from the chest and the chin moving downwards towards the chest, respectively). If the participant primarily experienced swaying (side-to-side motion sensation), then the participant’s head was manually moved side to side, resulting in the same movements produced by lateral flexion of the cervical vertebrae (i.e. left ear moving towards the left shoulder and right ear towards the right shoulder). If the participant primarily experienced rocking (forward and back motion sensation), then the participant’s head was manually moved side-to-side as per swaying, as passive protraction and retraction of the head while seated is often uncomfortable. This approach is consistent with previous literature [20, 21, 25].

### Statistical analysis

Continuous baseline demographic data were analysed using independent sample *t*-tests to assess pre-intervention similarity between groups and the effectiveness of the randomization process. Categorical baseline data were compared using Chi-square analysis (Fisher’s exact test) and independent samples *t*-tests. To determine the effect of active iTBS when compared to sham, subjective measures (MdDS symptom rating, mental health scores and disability perception) and objective assessments (posturography outcomes) were analysed using mixed-model analyses of variance (ANOVAs) with between-subject factor ‘Group’ (active vs. sham) and within-subject ‘Time’ (*MdDS symptom rating*: pre-treatment average, Day 1, Day 2, Day 3, Day 4, and 1-, 2-, 3-, 4-, 16-week post-treatment, *Mental health scores and disability perception*: Day 1, Day 4, 4- and 16-week post-treatment, Posturography: Day 1 baseline, and end of Days 1, 2, 3 and 4). Where appropriate, post-hoc analyses were performed using Sidak-adjusted multiple comparison tests. Assumptions of normality and sphericity were assessed using the Shapiro–Wilk test and Mauchly test of sphericity, respectively. The Greenhouse–Geisser correction for non-sphericity was applied for data sets that violated the assumption of sphericity. Statistical significance was set at *p* < 0.05. Partial eta squared (*η*^2^) is reported for all significant results. All data are presented in mean ± standard deviation (SD) unless otherwise indicated. Due to the small proportion of NMT participants compared to MT participants, statistical analysis was not performed between the onset groups.

#### Sample size calculations

No previous studies have evaluated the effectiveness of a combined TBS/VOR intervention for the management of symptoms associated with MdDS. An exploratory target sample of 20 participants was therefore employed, which is consistent with previous studies that have demonstrated significant effects of non-invasive brain stimulation protocols for the treatment of MdDS [33–37, 50, 57, 79–81].

## Results

### Demographics

Twenty-one participants were recruited into the clinical trial. The participants were randomly allocated to one treatment group (Active or Sham). One female MT participant (Active Group) withdrew from the study before completing the treatment due to a migraine that began the day before the trial commenced. As the data set was incomplete, the participant’s data are not included in the analysis presented in this manuscript.

The random group allocation resulted in a homogenous dataset, with no significant differences between the demographics (i.e., sex, onset type, age, duration of MdDS, resting motor threshold, various baseline rating/scores, motion symptom experience, menopause experience and diagnosed conditions) of the two treatment groups (Table 2). The majority of participants were female, which is consistent with MdDS prevalence data [3–6, 13, 16, 20, 33, 50]. The number of participants who correctly guessed their group allocation (60%) was no more than would be expected due to chance, indicating that participant blinding was successful.

**Table 2:** Participant demographics

	Active group	Sham group	*p *value
*N*	10	10	–
Sex (m/f)	1:9	0:10	*–*
Onset	2 NMT, 8 MT	1 NMT, 9 MT	*–*
Age in years	51.0 (12.4)	47.0 (9.8)	0.425
Right-handed	9	10	–
Duration of MdDS (months)	43.5 (26.7)	60 (45.6)	0.340
Baseline symptom rating/10	3.2 (1.3)	3.6 (1.5)	0.495
Baseline BDI	11.0 (2.3)	13.8 (2.2)	0.383
Baseline HADS—Anxiety	7.8 (4.7)	10.6 (4.4)	0.184
Baseline HADS—Depression	6.8 (5.0)	7.8 (3.5)	0.608
Baseline DHI	44.8 (21.9)	45.0 (18.1)	0.982
Rocking	70%	60%	1.000
Swaying	70%	90%	0.582
Bobbing	70%	80%	1.000
Menopause	44%	20%	0.350
Resting motor threshold (%MSO)	53 (5)%	52 (8)%	0.762
Diagnosed comorbidities (%group)			
Mitral valve prolapse (mild)	0%	10%	–
Frequent headache	40%	40%	–
Migraine	10%	10%	–
Anxiety	10%	20%	–
Vestibular migraine	10%	10%	–
High blood pressure	20%	10%	–
Athlete’s heart	0%	10%	–
Hypothyroidism	0%	10%	–
Pituitary adenoma	0%	10%	–
Post-traumatic stress disorder	10%	0%	–
Gastro-oesophageal reflux disease	10%	0%	–
Diabetes	10%	0%	–

Data are presented as mean (SD) unless otherwise indicated

*MT*  motion-triggered, *NMT* non-motion-triggered, *F* female, *M* male, *SD* standard deviation

### MdDS symptom rating

**Fig. 1:**
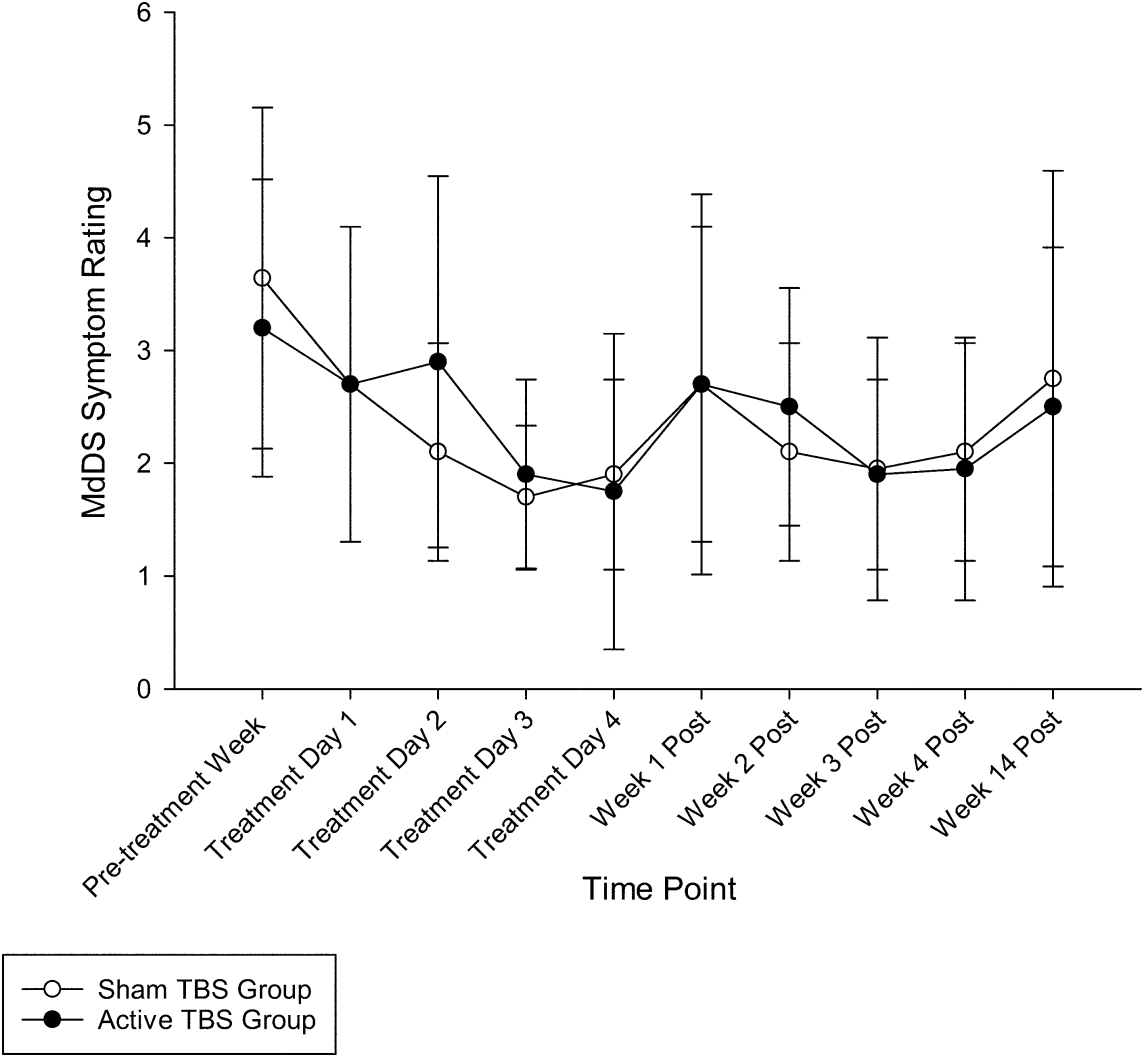
MdDS symptom rating data over time. Data are presented as mean (SD) unless otherwise indicated. Legend: *TBS* theta burst stimulation

At baseline, the MdDS symptom rating for the Active Group and the Sham Group fell within the 3–4 range (*Rocking/bobbing/swaying sensation is almost constant but can function fairly well with occasional rest periods)* according the MdDS Symptom Severity scale (Table 2). During the treatment week and at post-treatment time points, both groups demonstrated comparable reductions in MdDS scores over time (*F*(9,162) = 4.254, *p* < 0.001, *η*^2^ = 0.191) (Fig. 1). However, there were no significant differences between the active and sham groups (Group: *F*(1, 18) = 0.013, *p* = 0.911; Group × Time: *F*(9,162) = 0.513, *p* = 0.863).

### Mental health scores

#### Beck depression inventory (BDI)

**Fig. 2:**
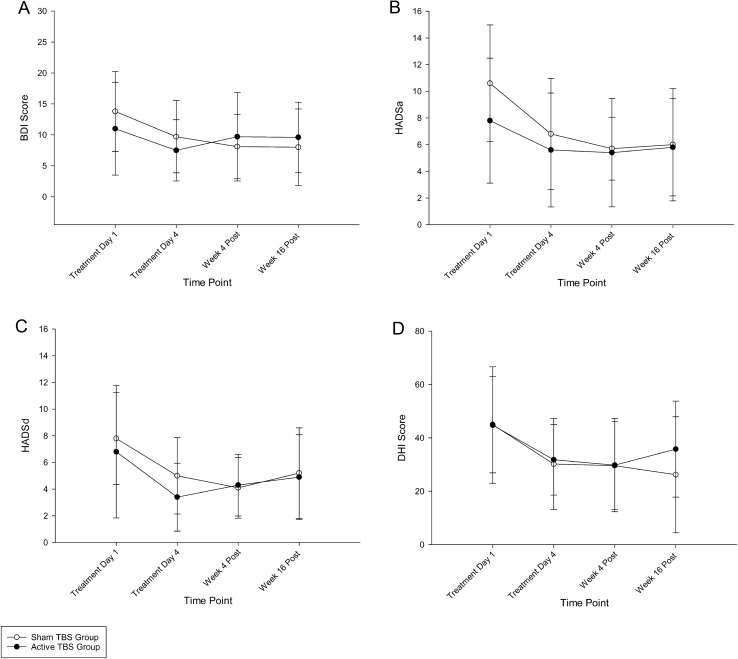
Mental health outcomes over time. **A** Beck depression inventory (BDI), **B** and **C** Hospital Anxiety and Depression Score – Anxiety and Depression (HADSa and HADSd) and D Dizziness Handicap Inventory (DHI) score. Data are presented as mean (SD) unless otherwise indicated. Legend: *TBS* theta burst stimulation

At baseline, the BDI scores for the Active Group and the Sham Group fell within the “mild – moderate depression” range (Table 2). Both groups demonstrated comparable reductions in BDI scores over time (*F*(3, 54) = 4.164, *p* = 0.01, *η*^2^ = 0.188); however, there were no significant differences between the Active and Sham Groups in terms of BDI scores over time (Group: *F* (1,18) = 0.039, *p* = 0.846; Group × Time: *F* (3,54) = 1.778, *p* = 0.162) (Fig. 2A).

#### Hospital Anxiety and Depression Score (HADS)

At baseline, the HADS—Anxiety (HADSa) score for the Active Group and the Sham Group were within the “borderline abnormal” anxiety range. Both groups demonstrated comparable reductions in HADSa scores over time (*F*(3, 54) = 11.229, *p* < 0.001, *η*^2^ = 0.384), to the “normal” range, with no significant between-group effects observed (Group: *F*(1, 18) = 0.508, *p* = 0.485; Group × Time: *F* (3,54) = 1.442, *p* = 0.241) (Fig. 2B). The baseline HADS—Depression (HADSd) score for the Active Group was within the “normal” depression range within the “borderline abnormal” range for the Sham Group. Both groups demonstrated comparable reductions in HADSa scores over time (*F*(3,54) = 5.873, *p* = 0.002, *η*^2^ = 0.246). As with HADSa scores, HADSd scores demonstrated no differences between groups over time (Group: *F*(1,18) = 0.460, *p* = 0.506; Group × Time: *F* (3,54) = 0.426, *p* = 0.735) (Fig. 2C).

### Disability perception

At baseline, the total Dizziness Handicap Inventory (DHI) score for the Active Group and the Sham Group were within the “moderate handicap” range (i.e., 36–52 points). While both groups demonstrated reductions in disability perception over time (*F*(3, 54) = 9.201, *p* < 0.001, *η*^2^ = 0.338), there were no significant between-group differences observed (Group: *F*(1, 18) = 0.159, *p* = 0.694; Group × Time: *F* (3,54) = 0.940, *p* = 0.428) (Fig. 2D).

### Posturography outcomes

#### Confidence ellipse area (CEA)

Compared to baseline, both groups demonstrated significant reductions in CEA over time (*F*(1.173, 21.116) = 14.978, *p* < 0.001, *η*^2^ = 0.454). However, there were no significant between-group differences observed (Table 3).

**Table 3:** Posturography outcomes

Measure	Group	Baseline	End of day 1	End of day 2	End of day 3	End of day 4	Results
CEA (cm^2^)	Active	63.5 ± 56.7	22.8 ± 15.0	16.4 ± 11.8	9.6 ± 4.6	11.8 ± 11.0	*F*(1.173, 21.116) = 0.440, *p* = 0.545^*a*^ *F*(1, 18) = 0.012, *p* = 0.913^*b*^ *F*(1.173, 21.116) = 14.978, ***p* < 0.001**, *η*^2^ = 0.454^c^
	Sham	53.2 ± 44.8	26.1 ± 17.3	17.3 ± 13.9	15.9 ± 12.2	15.9 ± 18.1	
AuC_ML (cm^2^)	Active	3.0 ± 2.9	0.9 ± 0.8	0.7 ± 0.6	0.4 ± 0.3	0.5 ± 0.5	*F*(1.390, 25.014) = 0.950, *p* = 0.369^a^ *F*(1, 18) = 0.342, *p* = 0.566^b^ *F*(1.390, 25.014) = 10.864, ***p* = 0.001**, *η*^2^ = 0.376^c^
	Sham	2.4 ± 1.9	1.5 ± 1.1	0.8 ± 0.6	1.0 ± 1.0	1.0 ± 1.7	
AuC_AP (cm^2^)	Active	4.4 ± 3.6	1.9 ± 1.2	1.2 ± 0.8	0.8 ± 0.4	0.9 ± 0.8	*F*(1.169, 21.050) = 0.220, *p* = 0.682^a^ *F*(1, 18) = 0.008, *p* = 0.926^b^ *F*(1.169, 21.050) = 15.691, ***p* < 0.001**, *η*^2^ = 0.466^c^
	Sham	3.9 ± 3.8	1.7 ± 1.3	1.3 ± 1.1	1.0 ± 0.9	0.9 ± 0.7	
DIS (cm)	Active	412.4 ± 265.8	165.2 ± 49.7	166.8 ± 61.2	130.6 ± 38.4	153.7 ± 70.7	*F*(1.159, 20.866) = 1.814, *p* = 0.193^a^ *F*(1, 18) = 0.217, *p* = 0.647^b^ *F*(1.159, 20.866) = 15.074, ***p* < 0.001**, *η*^2^ = 0.456^*c*^
	Sham	293.7 ± 178.6	192.9 ± 51.0	159.0 ± 64.0	158.5 ± 56.5	153.4 ± 50.3	
VEL (cm/s)	Active	7.23 ± 4.36	3.23 ± 0.85	3.27 ± 1.02	2.68 ± 0.64	3.03 ± 1.18	*F*(1.173, 21.114) = 1.987, *p* = 0.173^a^ *F*(1, 18) = 0.004, *p* = 0.948^b^ *F*(1.173, 21.114) = 14.124, ***p* < 0.001**, *η*^2^ = 0.440^*c*^
	Sham	5.40 ± 2.84	3.86 ± 0.88	3.38 ± 1.04	3.37 ± 0.92	3.28 ± 0.81	

Significant p values are in bold

Improvements were observed across all posturography outcomes over time, though there was no significant difference between the two groups over time

*CEA*  Confidence Ellipse Area, *AUC_ML* Area Under the Curve – Medial/Lateral, *AuC_AP*  Area Under the Curve – Anterior/Posterior, *DIS* distance, *VEL* velocity

^a^Tests of Within-Subjects Effects (Time*Group) – Greenhouse–Geisser

^b^Tests of Between-Subjects Effects (Group)

^c^Tests of Within-Subjects Effects (Time) – Greenhouse–Geisser, *η*^2^ = Partial Eta Squared

#### Area under the curve – medial/lateral (AuC_ML) and anterior/posterior (AuC_AP)

Both groups demonstrated significant reductions in AuC_ML (*F*(1.390, 25.014) = 10.864, *p* = 0.001, *η*^2^ = 0.376) and AuC_AP (*F*(1.169, 21.050) = 15.691, *p* < 0.001, *η*^2^ = 0.466) over time. However, there were no significant between-group differences observed for either variable (Table 3).

#### Distance (DIS)

When compared to baseline, both groups demonstrated reductions in DIS over time (*F*(1.159, 20.866) = 15.074, *p* < 0.001, *η*^2^ = 0.456). However, there were no significant between-group differences observed (Table 3).

#### Velocity (VEL)

While both groups demonstrated reductions in VEL over time (*F*(1.173, 21.114) = 14.124, *p* < 0.001, *η*^2^ = 0.440), there were no significant between-group differences observed (Table 3).

### Other reports

Only three sessions out of around 300 (~ 0.01%) VOR sessions were stopped prematurely due to issues with light sensitivity. On occasion, some participants received less than five sessions on Day 2 (15%), Day 3 (25%), or Day 4 (25%), due to these participants reporting significant improvements (or complete resolution) in their symptoms following the preceding session of VOR.

## Discussion

This study was the first to explore the synergistic effects of iTBS and the VOR rehabilitation protocol in people with MdDS. Despite significant improvements in subjective and objectives outcomes over time, there were no differences between the Active and Sham iTBS Groups. In both groups, MdDS symptom rating, mental health scores and disability perception significantly decreased, and effects were maintained up to the 16 weeks post-treatment follow-up. Both Sham and Active Groups demonstrated significant improvements in static posturography, with ~ 58–83% reductions across all outcomes by the end of treatment week. The results of this study suggests that iTBS of the DLPFC does not enhance outcomes beyond that achieved using the VOR protocol in the treatment of people with MdDS.

Over the last decade, various forms of non-invasive brain stimulation have been trialled on the MdDS population, such as rTMS [33–37], TBS [50], Transcranial Alternating Current Stimulation (tACS) [79–81], and Transcranial Direct Current Stimulation (tDCS) [57], and have demonstrated positive results. This is the first study that has trialled iTBS in this population and was chosen as the pre-treatment due to its potential to enhance synaptic plasticity, promote synaptogenesis, and facilitate synaptic connections within cortical tissue [82–85], with the aim to enhance the brain’s responsiveness for subsequent treatments. Further, iTBS over DLPFC has previously been shown to enhance postural control among other population groups [86]. iTBS also requires less session time compared to rTMS protocols [49]. Cha and colleagues [50] have conducted the only other study utilising TBS in MdDS, where continuous TBS (cTBS) was administered over the occipital cortices, cerebellar vermi, and lateral cerebellar hemispheres of 26 patients. The participants then had the freedom to continue receiving cTBS over the brain targets of their own preference, which they felt were most effective in reducing their oscillating vertigo. After the first session, eleven participants chose the occipital cortex, nine chose the cerebellar vermis, one chose lateral cerebellar hemisphere, and five chose none. After 10–12 sessions of 1200 pulses over the target of choice, it was concluded that cTBS over either the occipital cortex or cerebellar vermis was effective in reducing subjective perception of oscillating vertigo acutely, improving mental health scores and reducing perceptions of disability. Improvements in objective balance were reported across all groups. While valuable, it is difficult to determine the mechanisms underlying the effects observed in the previous study given that multiple sites were stimulated and there was the potential for compound effects between the initial TBS session and the participant’s selected target site. When combined with the findings of the present study, it is plausible that no single optimal cortical target site exists for MdDS, but rather, may vary between patients depending on their predominant motion symptom experience, triggers, or underlying pathogenesis [3, 5, 6, 13].

There are various potential reasons as to why iTBS over DLPFC did not augment the effectiveness of the VOR protocol. First, previous work demonstrates that the effects of non-invasive brain stimulation are cumulative, increasing with repeated sessions [87]. Therefore, while ecologically valid and more feasible clinically, the 4-day protocol employed in the present study may not have been sufficient to induce observable changes beyond those achieved with the VOR rehabilitation protocol alone. However, this remains speculative and it should be noted that a recent paper identified no cumulative effects of TBS over DLPFC on cortical excitability [88]. Second, the current study utilised an excitatory iTBS protocol, based on the positive effects observed in people with MdDS after excitatory rTMS over left DLPFC [33, 34]. iTBS over DLPFC has been shown to improve postural control in other population groups [86], though has not been trialled in people with MdDS. The goal of the protocol used in this study was to decrease functional connectivity between the entorhinal cortex and the posterior default mode network, in accordance with the findings of Yuan and colleagues [41], where positive outcomes were associated with a decrease in functional connectivity after excitatory rTMS over the left DLPFC. However, the DLPFC has far reaching connectivity with other cortical and subcortical sites, which may elicit varying or competing effects via indirect stimulation. Indeed, iTBS over remote and interconnected cortical sites has been shown to *enhance* functional connectivity in the default mode network, which may worsen MdDS symptoms [89]. Though incompletely explored, there is also the potential that the effectiveness of neuromodulation protocols varies depending upon patient presentation. For example, while TBS has been shown to be comparable or superior to rTMS for depression [49, 90], studies have suggested that high-frequency rTMS is superior to TBS for neuropathic pain [91]. Finally, there is a substantial body of literature suggesting that rTMS [92, 93] and iTBS [94, 95] induce variable effects on cortical excitability, with ‘excitatory’ and ‘inhibitory’ protocol labels being a misnomer. Inter-individual variability in cortical responses may therefore have ‘washed out’ effects during group-level analyses. Exploration of participant-specific responses in larger samples would be an interesting and important avenue for future research.

Recently, Mucci and colleagues postulated that MdDS originates from the persistence of an adaptive internal model that functions to cancel sinusoidal disturbances of body position experienced aboard a vehicle in motion [96]. It was proposed that the internal model is a bilateral oscillator, consisting of a system of loops, involving glutamatergic and GABAergic pathways between the cerebellar cortex and the vestibular nuclei in the brainstem. This vestibulo-cerebellar oscillator then becomes noxiously permanent in those with some sort of predisposing factor (i.e. immunoendocrine condition or disruption). A computational analysis of this proposed loop was investigated by Burlando et al. [97] and showed that parameter changes, typically induced by synaptic plasticity, increased the system’s tendency to oscillate. The results of this study may suggest that iTBS over the left DLPFC is not effective in disrupting the system of loops that are theorised to exist between the vestibular nuclei and the cerebellum, whereas cTBS over the cerebellum may be able to affect the noxious oscillator as evidenced by the positive effects of this approach observed in previous research [50].

Regardless of the result that iTBS of the left DLPFC does not enhance outcomes beyond that achieved using the VOR protocol in the treatment of people with MdDS, our study further validates the VOR rehabilitation protocol as an effective treatment option. The exact mechanism by which the VOR rehabilitation protocol produces beneficial outcomes in MdDS patients is not fully understood. In light of the recent vestibulo-cerebellar oscillator theory, it may be possible that aspects of the treatment influence these noxious loops between the cerebellum and the brainstem. The visuomotor functions of the cerebellum include control of the VOR and optokinetic reflexive eye movements, and smooth pursuit [98], all of which are activated in the VOR rehabilitation protocol. In addition, the passive movement of the vestibular apparatus via head movement could lead to an increase in peripheral afferent signals arriving at the vestibular nuclei, into the loop, potentially disrupting or weakening it. Though it is still not considered a cure, the VOR rehabilitation protocol has the capacity to improve subjective and objective outcomes in people with MdDS up to sixteen weeks post-treatment. These results are in line with the findings of Dai [21, 25], and Mucci [20], whereby ~ 70% of patients demonstrated significant improvements in objective and subjective outcomes after the VOR treatment. Dai [25] and Mucci [20] both reported that people with MT MdDS responded better to the treatment than those with NMT MdDS, and a differing underlying pathological mechanism between the onset types has been proposed [3, 5, 6, 13]. Given the small sample size of this study, the comparison between MT and NMT participants was not made. In addition to the unequal response rates, remission is rare and residual symptoms remain. This highlights that though the VOR rehabilitation protocol is the most effective treatment for MdDS, there is a need to further explore how the protocol can be optimised as part of MdDS management.

## Limitations

The main limitation to this study was not collecting objective posturography data at the 16-week follow-up timepoint. Given the geographical location of the participants, obtaining this data was not possible, but may have provided further insight into any delayed or long-term effects. This is a pertinent consideration given that the effects of non-invasive brain stimulation are often delayed beyond the treatment session itself [87]. Another limitation of the study was that our sample was exploratory and inter-individual variability was not analysed. Due to the inability to administer an effective sham version of the VOR rehabilitation protocol, a third group, i.e. active iTBS + sham VOR protocol, was not included in this study. This may be viewed as a limitation, though a sham VOR rehabilitation protocol would be difficult to blind. Another limitation is that some of the participants received their diagnosis from a Primary Care Physician, as opposed to a vestibular specialist. Given that MdDS is relatively uncommon and not investigated widely amongst Primary Care Physicians, this consideration may have influenced the population recruited (though also enabled larger samples to be recruited). However, most participants were reviewed by specialists, and all were required to be reporting symptoms consistent with MdDS diagnosis guidelines [3]. Finally, participant-specific MRI images were not employed during neuronavigation to localise DLPFC for stimulation. While the BeamF3 heuristic utilised has been shown to produce a very close approximation of the scalp site used for MRI-guided stimulation [99], there is the potential that participant-specific MRI data may have further enhanced target site localisation, and the use of this approach represents an important consideration for further research.

## Conclusion

This study demonstrates that a pre-treatment of iTBS of the left DLPFC does not enhance subjective or objective outcomes beyond that achieved using the VOR rehabilitation protocol in the treatment of people with MdDS. These findings further support the effectiveness of the VOR rehabilitation protocol in reducing MdDS symptoms. Though pretreatment of iTBS did not affect these improvements, further research into TBS efficacy is warranted, given the accumulating evidence in its ability to alter central processes in a non-invasive, non-pharmacological way, reduce MdDS symptoms when other areas of the brain are targeted and its potential to influence noxious loops between the cerebellum and the brainstem, which are theorised to play a vital role in MdDS.

## Data Availability

The original contributions presented in the study are included in the article, further inquiries can be directed to the corresponding authors.
